# Supplementary material for: Mechanisms governing the pioneering and redistribution capabilities of the non-classical pioneer PU.1
Source: Nat Commun. 2020 Jan 21;11:402. doi: 10.1038/s41467-019-13960-2 (PMC6972792; doi:10.1038/s41467-019-13960-2)
Supplement: Supplementary file 7 — Source data [file 41467_2019_13960_MOESM7_ESM.zip › Source_Data/Figure5/Figure5A_MotifScanOutput/homerResults/motif31.similar.html]

motif31

## Information for motif31

A
C
G
T
A
C
T
G
A
G
T
C
C
G
A
T
C
G
T
A
C
A
T
G
T
A
G
C
C
G
T
A
C
T
G
A
A
G
C
T
A
T
G
C
C
G
T
A
  
Reverse Opposite:  

A
C
G
T
T
A
C
G
C
T
G
A
A
G
C
T
A
C
G
T
A
C
T
G
G
T
A
C
C
G
A
T
C
G
T
A
A
C
T
G
A
G
T
C
C
G
T
A
  

|  |  |
| --- | --- |
| p-value: | 1e-41 |
| log p-value: | -9.560e+01 |
| Information Content per bp: | 1.929 |
| Number of Target Sequences with motif | 70.0 |
| Percentage of Target Sequences with motif | 2.32% |
| Number of Background Sequences with motif | 87.5 |
| Percentage of Background Sequences with motif | 0.19% |
| Average Position of motif in Targets | 186.6 +/- 153.2bp |
| Average Position of motif in Background | 210.5 +/- 146.4bp |
| Strand Bias (log2 ratio + to - strand density) | 0.2 |
| Multiplicity (# of sites on avg that occur together) | 1.00 |
| Motif File: | file (matrix) reverse opposite |

### Similar de novo motifs found

|  |  |  |  |  |  |  |  |
| --- | --- | --- | --- | --- | --- | --- | --- |
| Rank | Match Score | Redundant Motif | P-value | log P-value | % of Targets | % of Background | Motif file |
| 1 | 0.866 | A C T G A G T C A C G T C G T A A C T G A G T C C G T A C G T A A G T C A T G C | 1e-24 | -55.295307 | 1.86% | 0.27% | motif file (matrix) |
| 2 | 0.853 | A C G T G T C A A C T G A G T C C G T A C G T A A C G T A T G C C T G A | 1e-19 | -45.457599 | 2.39% | 0.58% | motif file (matrix) |
| 3 | 0.773 | A G C T A C T G A G T C A C G T C G T A A C T G A G T C | 1e-12 | -29.473396 | 5.17% | 2.66% | motif file (matrix) |
